# 369. The DOOR is open: A Web-based Application for Analyzing the Desirability of Outcome Ranking

**DOI:** 10.1093/ofid/ofad500.439

**Published:** 2023-11-27

**Authors:** Toshimitsu Hamasaki, Yijie He, Scott R Evans

**Affiliations:** The George Washington University, Rockville, Maryland; The George Washington University Biostatistics Center, Rockville, Maryland; Milken Institute School of Public Health, Rockville, Maryland

## Abstract

**Background:**

The Antibacterial Resistance Leadership Group (ARLG) has developed the Desirability of Outcome Ranking (DOOR) methodology, a novel paradigm for the design, conduct, analysis and interpretation of clinical trials. The DOOR methodology enables clinicians to effectively evaluate and select treatment strategies by providing an informative way to compare the patient-centered risks and benefits of intervention alternatives. There are two complementary approaches to DOOR analysis. The first approach summarizes DOOR responses between treatments by the probability of a more desirable outcome in one treatment relative to another (DOOR probability) using the concept of pairwise comparisons, The second, “partial credit” analyses, involves grading the DOOR levels on a scale from 0 (least desirable) to 100 (most desirable), allowing for strategic scoring, preferential assignment of relative importance to DOOR levels for personalized analyses, and evaluation of robustness through analysis of different scoring keys.

**Methods:**

The Statistical and Data Management Center of the ARLG is developing interactive web-based applications (apps) with downloadable data presentations, to help clinical researchers conduct DOOR analyses.

**Results:**

Two editions of the apps are available. The Standard Edition (Std) requires entry of summary level data of the DOOR outcome by intervention group. The app generates ARLG-recommended components of comprehensive DOOR analyses, including summary tables and figures of DOOR distributions, forest plot displays of DOOR probability estimates for the DOOR and its components, and partial credit analysis. The Professional Edition (Pro) reads patient-level data. In addition to the analyses provided in the Std, the Pro includes additional functionality and informed visualizations, i.e., inverse probability weighting (IPW)-based analyses, tie-breaker analyses, and the Anthology of Patient Stories plot.

Data Entry Panel of the Application (Standard Edition)

**Figure:**
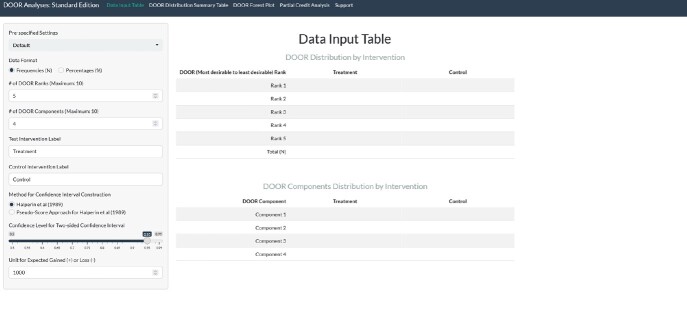

DOOR Frorst Plot Output (Standard Edition)

**Figure:**
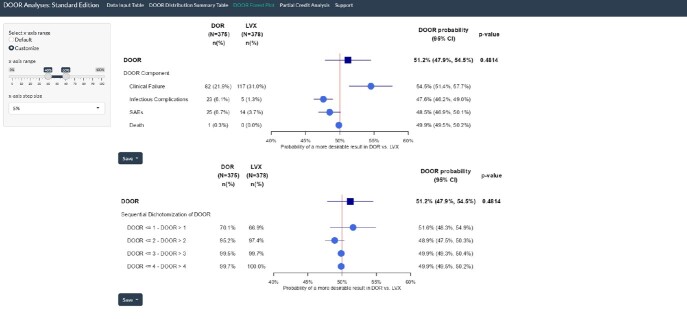

DOOR Forest Plot Output from DORI-05 study of comparing IV doripenem (DOR) to IV levofloxacin (LVX), with a step-down option of oral levofloxacin in both groups after 3 days of IV therapy (Standard Edition)

**Figure:**
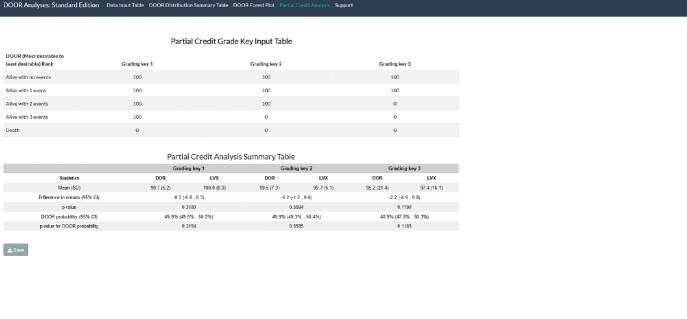

Partial credit analysis output from DORI-05 study of comparing IV doripenem (DOR) to IV levofloxacin (LVX), with a step-down option of oral levofloxacin in both groups after 3 days of IV therapy (Standard Edition)

**Conclusion:**

The DOOR apps provide comprehensive tools for researchers to implement the DOOR methodology. The apps are freely available online at https://methods.bsc.gwu.edu/.

**Disclosures:**

**All Authors**: No reported disclosures

